# Arrhythmia Diagnosis Following an ICD Shock: Comment

**Published:** 2002-01-01

**Authors:** Pual A Levine

This case study by Dr. John [1] demonstrates the care that one must take in programming an implantable cardioverter defibrillator, the complexity of these patients and the utility of the internal diagnostics in the system to facilitate the physician's understanding of device behavior. Even the single chamber ICDs have SVT discrimination algorithms that can be enabled in an effort to differentiate SVT from VT allowing the device to withhold ATP and shock therapy in the setting of an SVT. Many physicians, myself included, will initially utilize these discriminators in a monitoring mode preferring to deliver unnecessary therapy rather than inappropriately withholding needed therapy. This also provides an opportunity to better understand all the rhythms that may be occurring in a patient, many of which may not have been appreciated prior to the implant. After one or more SVT episodes have occurred and the clinician has a chance to review the stored electrograms (first introduced by Ventritex) along with the response of the device to any discriminators, a decision can be made as to how the ICD prescription might be adjusted.

My  examination of the stored electrogram documenting the initial atrial fibrillation episode suggests significant irregularity of the ventricular response. As such, had the stability criteria been enabled, it is likely that the system would have appropriately recognized the rhythm as atrial fibrillation and withheld therapy. If the stability criteria had been programmed to the monitoring mode, the retrieved information would have indicated that the diagnosis was supraventricular but therapy was delivered because the discriminator was not active. A further refinement in single chamber and more recent dual chamber ICDs is a feature called morphology discrimination. This would allow the device to separate a rapid irregular ventricular response falling within the VT rate zone associated with atrial fibrillation from polymorphic ventricular tachycardia. Given the complexity of these patients, if atrial fibrillation was hemodynamically compromising, it might be appropriate to not enable any discrimination algorithms. Dr. John indicates that amiodarone was not tolerated and other drugs have both a higher incidence of side effects and lower efficacy rate. If the episodes of atrial fibrillation were not frequent, another option would be to allow the AF to trigger ATP therapy which was arrhythmogenic but then take advantage of the subsequent shock to not only terminated the induced-VT but also terminate the atrial fibrillation as occurred in this case.
